# Salivary orosomucoid 1 as a biomarker of hepatitis B associated hepatocellular carcinoma

**DOI:** 10.1038/s41598-022-18894-2

**Published:** 2022-09-12

**Authors:** Jiaoxia He, Zhongling Zheng, Tingting Liu, Yupei Ao, Yixuan Yang, Huaidong Hu

**Affiliations:** Department of Infectious Diseases, Key Laboratory of Molecular Biology for Infectious Diseases (Ministry of Education), Institute for Viral Hepatitis, The Second Affiliated Hospital, Chongqing Medical University, Chongqing, 400000 People’s Republic of China

**Keywords:** Tumour biomarkers, Cancer screening

## Abstract

Saliva is rich in proteins, DNA, RNA and microorganisms, and can be regarded as a biomarker library. In order to explore a noninvasive and simple means of early screening for liver cancer, proteomics was used to screen salivary markers of hepatitis B associated liver cancer. We used mass spectrometry coupled isobaric tags for relative and absolute quantitation (iTRAQ)-technology to identify differentially expressed proteins (DEPs). Western blot, immunohistochemistry and enzyme linked immunosorbent assay were used to detect marker expression of in tissues and saliva. Statistical analysis was used to analyze the diagnostic efficacy of the markers was analyzed through statistical analyses. By comparing the hepatocellular carcinoma (HCC) group with non-HCC groups, we screened out 152 salivary DEPs. We found orosomucoid 1(ORM1) had significantly higher expression in saliva of HCC patients compared with non-HCC groups (*p* < 0.001) and the expression of ORM1 in liver cancer tissues was significantly higher than that in adjacent normal tissues (*p* < 0.001). The combination of salivary ORM1 and alpha-fetoprotein (AFP) showed reasonable specificities and sensitivities for detecting HCC. In a word, salivary ORM1 as a new biomarker of hepatitis B associated hepatocellular carcinoma, combination of salivary ORM1 and AFP as an improved diagnostic tool for hepatocellular carcinoma.

## Introduction

According to the International Agency for Research on Cancer, cancer of the liver ranks sixth in terms of incidence, fifth in men and ninth in women, and ranks third as the cause of cancer-related deaths^1^. Hepatocellular carcinoma (HCC) is the most common pathological type of liver cancer, representing 90% of cases^2^. Hepatitis B virus (HBV) infection is the most important risk factor for HCC^3^.The incidence of liver cancer has declined over the past decade, but the fatality rate remains high^1^. The 5-year survival rate of early liver cancer can be greatly improved, compared with advanced liver cancer, via effective treatment, and early diagnosis is especially important to improve the prognosis of liver cancer^4^. Early screening and monitoring can be used as an independent factor affecting the prognosis of HCC^5^. At present, b-ultrasound and serum alpha-fetoprotein are commonly used for screening and detecting liver cancer. B-ultrasound detection requires at least 8 h of fasting, and the accuracy of the detection is limited by liver characteristics (such as abnormal liver texture), patient characteristics (such as obesity), and technical limitations (such as ultrasound quality and experience)^6^. Compared with ultrasound, biomarkers used to screen tumors are more convenient for both doctors and patients. The current biomarker commonly used to screen for liver cancer is AFP. The sensitivity of ultrasound for early liver cancer screening was 47% (95%CI: 33–61%), and that of ultrasound combined with AFP for early liver cancer screening was 63% (95%CI: 48–75%)^4^. With technological advances, AFP-L3%, DES-γ -carboxy prothrombin (DCP) and other biomarkers and liquid biopsy techniques can increase the sensitivity and specificity of liver cancer detection and achieve good results^7^.

Saliva is a slightly acidic oral fluid, composed of salivary gland secretions, gingival crevicular fluid, and serum exudate microorganisms. It is rich in protein and cell components and can be used for early screening and monitoring of various types of cancers^8^. It has been reported that saliva LNC-PCDH9-13:1 is an ideal biomarker for early HCC^9^. A model of 12 salivary metabolites had a sensitivity of 84.8% and a specificity of 92.4% for the diagnosis of HCC^10^.

Isobaric tags for relative and absolute quantitation (iTRAQ)-based mass spectrometry has been widely used in the research of various diseases^11^. Saliva detection has the advantages of being non-invasive and a relatively simple procedure. We used proteomics methods based on iTRAQ to reveal the differences in salivary protein levels between liver cancer patients and healthy, cirrhotic, and viral hepatitis B patients with the goal of identifying potential salivary markers of liver cancer.

## Methods and materials

### Subject selection

The study was approved by the Ethics Committee of the Second Affiliated Hospital of Chongqing Medical University (Grant no. 28/2021). Written informed consent was obtained from all participants before participating in the present study. All methods were performed in accordance with the relevant guidelines and regulations. Subjects were recruited from the Department of Infectious Diseases and the Physical Examination Center of the Second Affiliated Hospital of Chongqing Medical University between April 1, 2021 and October 31, 2021. This study was performed at the Second Affiliated Hospital of Chongqing Medical University. This study included discovery and validation cohorts. Each cohort included HCC patients, liver cirrhosis (LC) patients, Chronic hepatitis B(CHB) patients, and healthy subjects(NC). Healthy subjects were individuals with normal health examination results, including chest X-ray, oral examination, abdominal ultrasound, routine stool examination, routine blood examination, liver and kidney function, HBV and HCV antigen test results, and no antibodies against HIV and syphilis. Chronic hepatitis B (CHB) was defined as chronic necroinflammatory liver function caused by persistent HBV infection (positive HBsAg over 6 months with serum HBV DNA > 10^5^ copies/ml and persistent or intermittent elevation in AST or ALT concentrations). Patients with LC were confirmed by biopsy or two imaging modalities (hepatic ultrasound with CT or MRI). HCC was diagnosed based on biopsy of the tumor or CT/MRI,and staged based on Barcelona Clinic Liver Cancer(BCLC) staging system^2^.We divided HCC into early HCC(BCLC 0- BCLC A) and advanced HCC(BCLC B- BCLC D).HCC patients were not treated with anticancer therapy. Viral hepatitis B was the cause of all liver cirrhosis and liver cancer patients in the study. Patients diagnosed with oral disease by a professional stomatologist were excluded. Patients with metabolic diseases, autoimmune diseases, cardiovascular and cerebrovascular diseases, respiratory disease and other cancers other than liver cancer were excluded.

### Saliva collection

Specimens were collected between 8 a.m. and 10 a.m. and subjects were required to avoid eating, drinking and smoking for at least 2 h before collection and to gargle with water immediately before collection^12^.We used the spitting method to collect whole saliva^13^, where the subjects spat saliva into pre-cooled 15 ml centrifuge tubes. The saliva sample was centrifuged at 4000 g for 15 min at 4 °C) to pellet shed cells. The supernatant was transferred to a 1.5 mL Eppendorf tube, followed by further centrifugation (12,000 g, 10 min, 4 °C) to completely remove the cell components. and add protease inhibitor was added to the samples (100:1) which were stored at − 80 °C until use.

### Salivary protein extraction and labeling

Ten saliva samples in each group of the discovery cohort were pooled together to obtain one mixed sample from each group. 4 groups of mixed samples were precipitated with cold acetone, and the protein concentration of the precipitated protein was detected by Bradford assay. Salivary proteins were suspended, denatured, cysteine blocked, and digested with trypsin. Each group of samples was labeled with iTRAQ marker reagent (Thermo Fisher Scientific, Inc., Waltham, MA, USA) and repeated with a different label (NC group was labeled 117 and 118; CHB was labeled 119 and 121; LC was labeled 115 and 116; HCC was labeled 113 and 114).

### Peptide fractionation

Liquid phase separation of the samples was performed with a Shimadzu LC-20AB liquid phase system on a 5um 4.6 × 250 mm Gemini C18 column. The elution peak was monitored at a wavelength of 214 nm and one component was collected per minute. The samples were combined according to the chromatographic elution peak map to obtain 20 fractions, which were then freeze-dried.

### High performance liquid chromatography

The dried peptide samples were reconstituted with mobile phase A (2% ACN, 0.1% FA), centrifuged at 20,000 g for 10 min, and the supernatant was taken for injection. Separation was performed on a Thermo UltiMate 3000 UHPLC (Thermo Fisher Scientific).

### Mass spectrometry detection

The peptides separated by liquid chromatography were ionized by a nanoESI source and passed to a Q-Exactive HF X tandem mass spectrometer (Thermo Fisher Scientific, Inc.) for Data Dependent Acquisition (DDA) mode detection. The ion fragmentation mode was HCD, and the fragment ions were detected via Orbitrap. ProteinPilot2.0 protein quantitative analysis software and the Uniprot database (http://www.uniprot.org/) were used for data processing. DEPs must have met the following conditions: 95% confidence interval and 5% false positive rate (FDR); protein identification requires at least 2 unique peptides; protein score selection threshold > 1.3^14^.

### Target protein determination

The top 10 up-regulated differentially expressed proteins(DEPs) were identified from the iTRAQ analysis, and were verified by the Western blot analysis of the mixed and individual saliva samples. Proteins that were identified by Western blot analysis as being significantly over-expressed in the liver cancer group were selected. Finally, proteins that were found, via immunohistochemistry(IHC) and enzyme-linked immunosorbent assay (ELISA), to be highly expressecd in liver cancer tissues and in the saliva of liver cancer patients were selected as the target proteins of this study.

### Western blot

The concentration of extracted proteins was determined via an Enhanced BCA Protein Assay kit (Beyotime Institute of Biotechnology, Haimen, China).The proteins of the 4 groups of mixed saliva samples in the discovery cohort were extracted and diluted with 5X loading buffer (Beyotime, China), then heated at 100 °C for 5 min to fully denature the proteins. Protein samples were subjected to 10% SDS-PAGE electrophoresis and transferred to a PVDF membrane (Millipore Corporation, Bedford, MA, USA).Blocked with 5% BSA and TBST for 2 h at room temperature. The membrane was then incubated with primary antibodies (all 1:1000; anti-AFP, ab169552, Abcam, UK; anti-orosomucoid1(ORM1), ab134042, Abcam, UK; anti-matrix metalloproteinase-9(MMP9), ab246539, Abcam, UK; anti-haptoglobin(HP), ab256454, Abcam, UK; anti-arginase1(ARG1), ab124917, Abcam, UK; anti-low affinity immunoglobulin gamma Fc region receptor III-B (FCGR3B), A7894, ABclonal, China;anti- coactosin-like protein (COTL1), A4550, ABclonal, China; anti-glyceraldehyde-3-phosphate dehydrogenase (GAPDH), 5174S, Cell Signaling Technology, America) at 4 °C for 12 h, and washed thrice with 1 × TBST. The membrane was incubated with HRP-IgG antibodies(1:5000;Santa Cruz Biotechnology, USA) at room temperature for 1 h, washed again with 1 × TBST. ECL reagents were prepared at a ratio of 1:1, and the bands were analysed using a ChemiDoc MP imaging system (Bio-Rad Laboratories).

### Immunohistochemistry

The expression of AFP, ORM1 and HP in HCC was detected via immunohistochemistry using an HCC tissue array (OD-CT-DgLiv03-003, Outdo Biotech,China), which included 31 HCC tissues and 31 matched normal adjacent tissues.The tissue chips were rehydrated in xylene and an ethanol gradient, washed with double distilled water and soaked in 3% H_2_O_2_ for 10 min to quench endogenous peroxidase activity. The tissue arrays were blocked with BSA for 30 min, and incubated with AFP, ORM1 and HP primary antibodies overnight at 4 °C. AFP, ORM1 and HP expression was measured using a DAKO EnVision + System, HRP (DakoCytomation, Glostrup, Denmark) under 400 × magnification. Nuclear staining intensity was classified as negative, weak, medium and strong, represented by the numbers 0,1,2,3 respectively. The number of positive cells was divided into five grades: 0, 1, 2, 3 and 4, indicating that the number of positive cells accounted for 0%, 1–25%, 26–50%, 51–75% and 76–100% of the total number of cells, respectively. Staining index (SI) was defined as the product of nuclear staining intensity score and positive cell number score (scope: 0–12).

### Elisa and experimental indicators were detected

The 200 saliva samples from the validation cohort were tested for AFP (ELH-AFP-1, Raybiotech, Peachtree Corners, GA, USA) and ORM1 (243,675, Abcam, UK) using ELISA kits. Serum AFP levels were determined by LIAISON automatic chemiluminescence analyzer (DiaSorin S.p.A., Italy). The measurement protocol was performed according to the instructions of the respective ELISA kits. Saliva and blood tests were performed independently by different researchers in two different laboratories. As the specimens were numbered randomly, the researchers did not know the basic information of the patients.

### Statistical analyses

All experiments were performed at least in triplicate. Statistical analyses were performed using SPSS 20.0 (IBM, Armonk, NY, USA). The quantitative variables of the normal distribution were represented as mean ± standard deviation (SD). The Student's-t test was used for comparison between the two groups, and analysis of variance was used for comparison between multiple groups. The quantitative variables of the non-normal distribution were represented by median (range), and the Mann–Whitney U test was used between two groups. Comparisons between multiple groups were analyzed using the Kruskal–Wallis H Test. For classification variables, we used the Chi-square test for differences between groups and Spearson correlation analysis to determine the correlation between two continuous variables (Saliva AFP and serum AFP). Receiver operating characteristic (ROC) curve analysis was used to evaluate the diagnostic performance of the target proteins. For the two markers combined diagnostic model we used the binary logistic regression model. P values less than 0.05 were considered to be statistically significant, and all significance tests were two-sided tests. Based on the area under the ROC curve, a type 1 error (α) of 5% and statistical power (1-β) of 90% (two-sided), we used Power Analysis and Sample Size (PASS) software to calculate the minimum sample size required.

### Ethics approval

The Present study was approved by the Ethics Committee of the Second Affiliated Hospital of Chongqing Medical University. All participants provided written informed consent prior to participation.

## Results

### Demographic and clinical characteristics of subjects

A total of 240 subjects were included in this study. There were 40 subjects in the discovery cohort, including 10 subjects in the HCC group, 10 subjects in the LC group, 10 subjects in the CHB group, and 10 subjects in the NC group (Table 1). There were no statistically significant differences in gender or age among the groups (*p* > 0.05). AST, ALT, AFP and HBV-DNA levels were different due to different disease states among all of the groups (*p* < 0.05). There were 200 subjects in the validation cohort, including 80 in the HCC group(40 patients with early HCC, 40 patients with advanced HCC), 40 in the LC group, 40 in the CHB group, and 40 in the NC group (Table 2). There were no statistically significant differences in age and gender among the groups (*p* > 0.05), and there were statistically significant differences in AST, ALT, AFP, and HBV-DNA levels among the groups (*p* < 0.05).

**Table 1:** Demographic and clinical characteristics of the discovery cohort.

Group	HCC	LC	CHB	NC	*p*
Number	10	10	10	10	
Age (year)	51.6 ± 7.95	52.9 ± 11.06	45.6 ± 11	49.4 ± 9.73	NS ^a,b,c^
Gender (male/female)	8/2	8/2	8/2	8/2	NS^a,b,c^
ALT (IU/ml)	63 (19,112)	22 (11,50)	1020 (160,1903)	22 (11,30)	*p* < 0.05 ^a,b,c^
AST (IU/ml)	109.5 (34,624)	34.5 (17,69)	443 (78,1237)	27.5 (17,37)	*p* < 0.05 ^a,b,c^
Serum AFP (ug/L)	860 (310,2260)	2.41 (0.69,16.5)	43.19 (2.97,452.8)	NA	*p* < 0.05 ^a,b,^
HBV-DNA (log IU/ml)	4.4(1.69,6.92)	1.69(1.69,1.69)	6.53(3.39,8.2)	NA	*p* < 0.05^a^, NS ^b^
HBeAg (Positive/negative) (*n*)	1/9	0/10	2/8	0/10	NS^a,b,c^
Early HCC (*n*)/advanced HCC (*n*)	5/5	NA	NA	NA	NA

The quantitative variables of the normal distribution were represented as mean ± standard deviation (SD), the student's-t test was used for comparison between the two groups, and analysis of variance was used for comparison between multiple groups. The quantitative variables of the non-normal distribution were represented by median (range), the Mann–Whitney U test was used between two groups. ^a^HCC group versus LC group; ^b^HCC group vserus CHB group; ^c^HCC group versus NC group. Abbreviations: *NC* normal control, *CHB* chronic hepatitis B, *LC* liver cirrhosis, *HCC* hepatocellular carcinoma, *ALT* alanine aminotransferase, *AFP* α-fetoprotein, *NA* Not Available, *NS* no significance.

**Table 2:** Demographic and clinical characteristics of the validation cohort.

Group	HCC	LC	CHB	NC	*p*
Number	80	40	40	40	
Age (year)	52.47 ± 8.65	51.93 ± 12.18	47.88 ± 9.36	48.2 ± 10.76	NS^a,b,c^
Gender (male/female) (n)	64/16	32/8	32/8	34/6	NS^a,b,c^
Serum ALT (IU/ml)	54.5 (13,533)	35 (11,1212)	428.5 (10,2466)	26 (11,41)	NS^a^, *p* < 0.05^b,c^
Serum AST (IU/ml)	81.95(20,558)	49 (15,1011)	190 (17,2134)	25.5 (13,38)	NS^a^, *p* < 0.05^b,c^
Serum AFP (ug/L)	301.7 (1.51,8890)	11.78 (1.63,358.3)	13.52 (1.55,600.6)	NA	*p* < 0.05^a,b^
HBV-DNA (log IU/ml)	3.11 (1.7,7.43)	1.7 (1.7,7.21)	5.24( 1.7,9.52)	NA	*p* < 0.05^a,b^
HBeAg (Positive/negative) (*n*)	23/57	9/31	21/19	0/40	NS^a^, *p* < 0.05^b,c^
Early HCC (*n*)/advanced HCC (*n*)	40/40	NA	NA	NA	

The quantitative variables of the normal distribution were represented as mean ± standard deviation (SD), the student's-t test was used for comparison between the two groups, and analysis of variance was used for comparison between multiple groups. The quantitative variables of the non-normal distribution were represented by median (range), the Mann–Whitney U test was used between two groups. ^a^HCC group versus LC group; ^b^HCC group versus CHB group; ^c^HCC group versus NC group. Abbreviations: *NC* normal control, *CHB* chronic hepatitis B, *LC* liver cirrhosis, *HCC* hepatocellular carcinoma, *ALT* alanine aminotransferase, *AFP* α-fetoprotein, *NA* Not Available, *NS* no significance.

### Saliva proteomes

By comparing the HCC group with the LC group, the CHB group and the NC group, 1623 DEPs were identified. There were 152 DEPs finding in all three comparison groups and meeting Uniq Peptide Number ≥ 2 and fold change > 1.3 or < 1/1.3,152 DEPs can be found as Supplementary Table S1 online. Comparing the HCC group to the non-HCC group, 41 DEPs were up-regulated and 111 were down-regulated. The top 10 up-regulated DEPs are shown in Table 3.

**Table 3:** The top 10 upregulated differentially expressed proteins.

Protein_ID(uniport)	Gene symbol	Description	Peptides (95%)	HCC versus NC (FC)	HCC versus CHB (FC)	HCC versus LC (FC)	Mean (FC)
Q9Y3C6	PPIL1	Peptidyl-prolyl cis–trans isomerase-like 1	2	2.43	2.51	2.28	2.41
P02771	AFP	Alpha-fetoprotein	11	2.53	2.47	2.19	2.39
O75015	FCGR3B	Low affinity immunoglobulin gamma Fc region receptor III-B	2	1.65	2.12	2.09	1.95
P00738	HP	Haptoglobin	9	1.35	2.22	2.19	1.92
P14780	MMP9	Matrix metalloproteinase-9	25	1.68	1.63	1.87	1.72
Q14019	COTL1	Coactosin-like protein	6	1.87	1.37	1.88	1.71
P05089	ARG1	Arginase-1	10	1.82	1.39	1.88	1.70
O43516	WIPF1	WASL-interacting protein family member 1	4	1.77	1.64	1.65	1.69
P02763	ORM1	Alpha-1-acid glycoprotein 1	5	1.33	1.68	1.86	1.62
P07737	PFN1	Profilin-1	14	1.76	1.41	1.68	1.62

Abbreviations: *NC* normal control, *CHB* chronic hepatitis B, *LC* liver cirrhosis, *HCC* hepatocellular carcinoma, *FC* Fold change.

### Target protein validation and determination

#### Western blot

**Figure 1:**
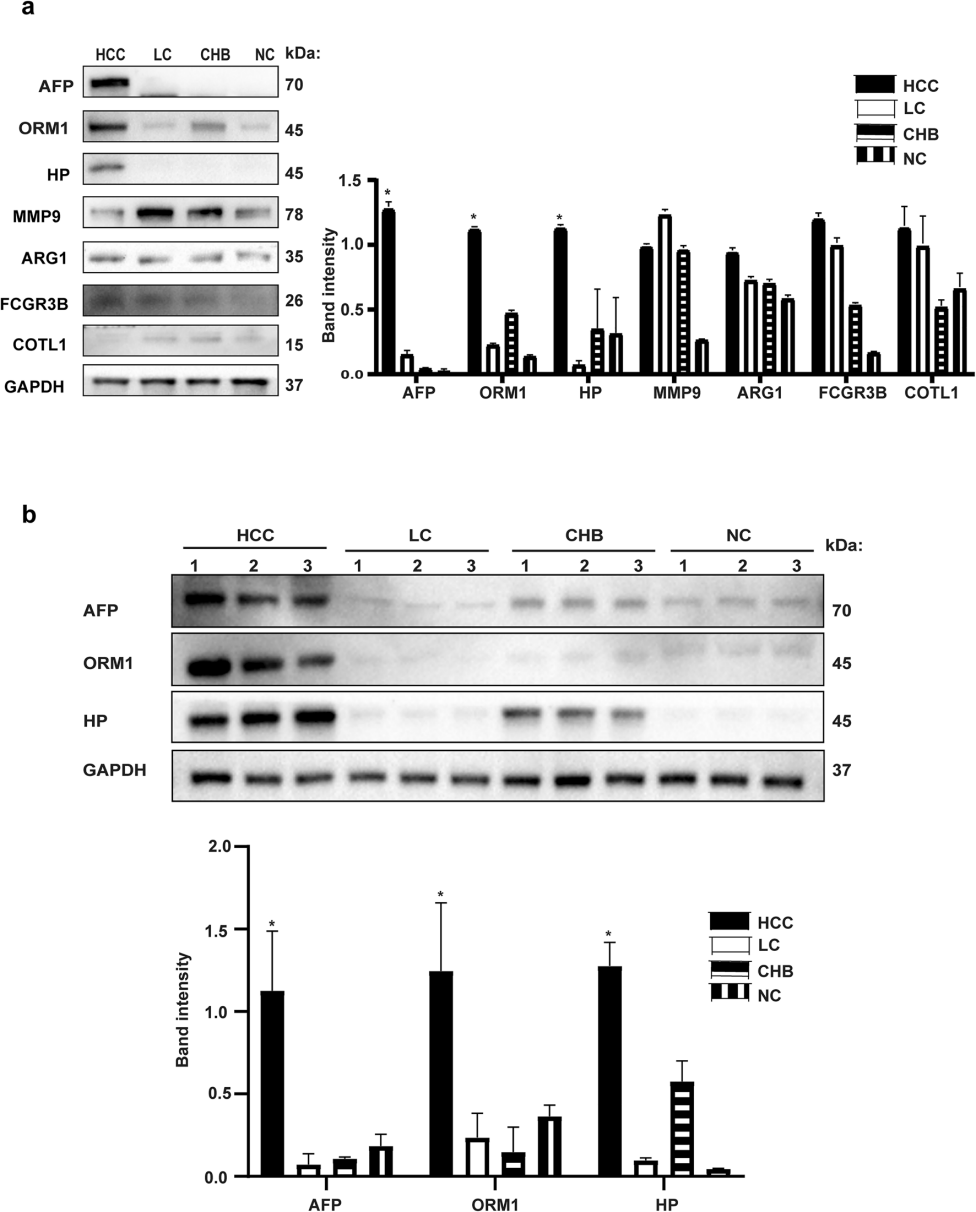
Western blot verification. (**a**) Representative images of Western blot for AFP, HP, ORM1, ARG1, COTL1, FCG3B and MMP9 in HCC, LC, CHB and NC mixed saliva; Analysis of band densities by Image J. (**b**) Representative images of Western blot for AFP, HP, and ORM1 in 3 subjects' saliva from HCC, 3 subjects' saliva from LC, 3 subjects' saliva from CHB and 3 subjects' saliva from NC; Analysis of band densities by Image J. The samples derive from the same experiment and blots were processed in parallel. Original blots are presented in Supplementary Figure. *HCC* hepatocellular carcinoma, *LC* liver cirrhosis, *CHB* chronic viral hepatitis B, *NC* normal control, *AFP* α-fetoprotein, *HP* Haptoglobin, *ORM1* Orosomucoid1, *ARG1* Arginase-1, *COTL1* Coactosin-like protein, *FCGR3B* Low affinity immunoglobulin gamma Fc region receptor III-B, *MMP9* Matrix metalloproteinase-9. Data are shown as mean ± SD, *p < 0.05, versus liver cirrhosis, chronic viral hepatitis and normal control.

According to the availability of antibody reagents for the top 10 up-regulated DEPs, we analyzed AFP, HP, ORM1, ARG1, COLT1, FCGR3B and MMP9 in the four groups of mixed saliva samples via Western blot. The expression of AFP, ORM1 and HP was significantly higher in the HCC group than in the non-HCC group (*p* < 0.05) (Fig. 1a). Saliva samples were randomly selected from 3 subjects in the discovery cohort, and these samples were also analyzed by Western blot to measure the expression levels of AFP, ORM1 and HP. AFP,ORM1 and HP were significantly higher in the HCC group than in the non-HCC group (*p* < 0.05) (Fig. 1b).

#### Immunohistochemistry

**Figure 2:**
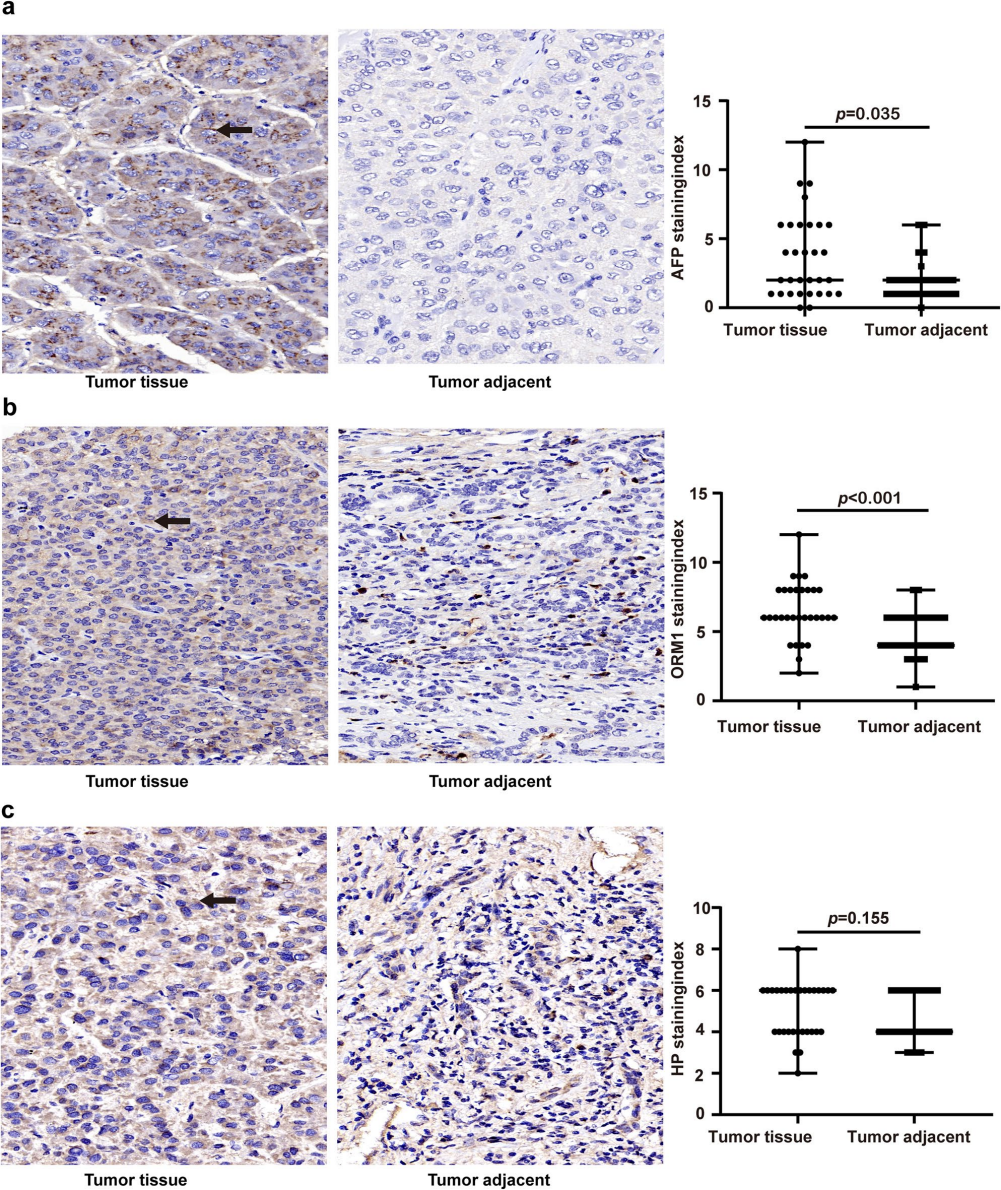
Immunohistochemistry verification. (**a**) Representative immunohistochemistry staining and analysis of differences in staining index of AFP in hepatocellular carcinoma tissue and tumor adjacent tissues. (**b**) Representative immunohistochemistry staining and analysis of differences in staining index of ORM1 in hepatocellular carcinoma tissue and tumor adjacent tissues. (**c**) Representative immunohistochemistry staining and analysis of differences in staining index of HP in hepatocellular carcinoma tissue and tumor adjacent tissues. AFP, ORM1 and HP are secreted proteins, localized in the cytoplasm and extracellular matrix. *AFP* α-fetoprotein, *HP* Haptoglobin, *ORM1* Orosomucoid1. Data are shown as median with range, *p < 0.05, **p < 0.001.

Three identical tissue chips,containing liver and normal adjacent tissues from 31 patients with hepatocellular carcinoma, were analyzed to compare the expression levels of AFP, ORM1 and HP. AFP and ORM1 showed strong staining in liver cancer tissues, and comparing the staining index of liver cancer tissue and normal adjacent tissue of the same patient, the expression of AFP and ORM1 in liver cancer tissue was significantly higher than that normal tissue (AFP: *p* = 0.035,ORM1:*p* < 0.001) (Fig. 2a and b). While HP showed no significant difference in staining intensity and the staining index between liver cancer tissues and adjacent normal tissues (*p* = 0.155) (Fig. 2c).

#### ELISA

**Figure 3:**
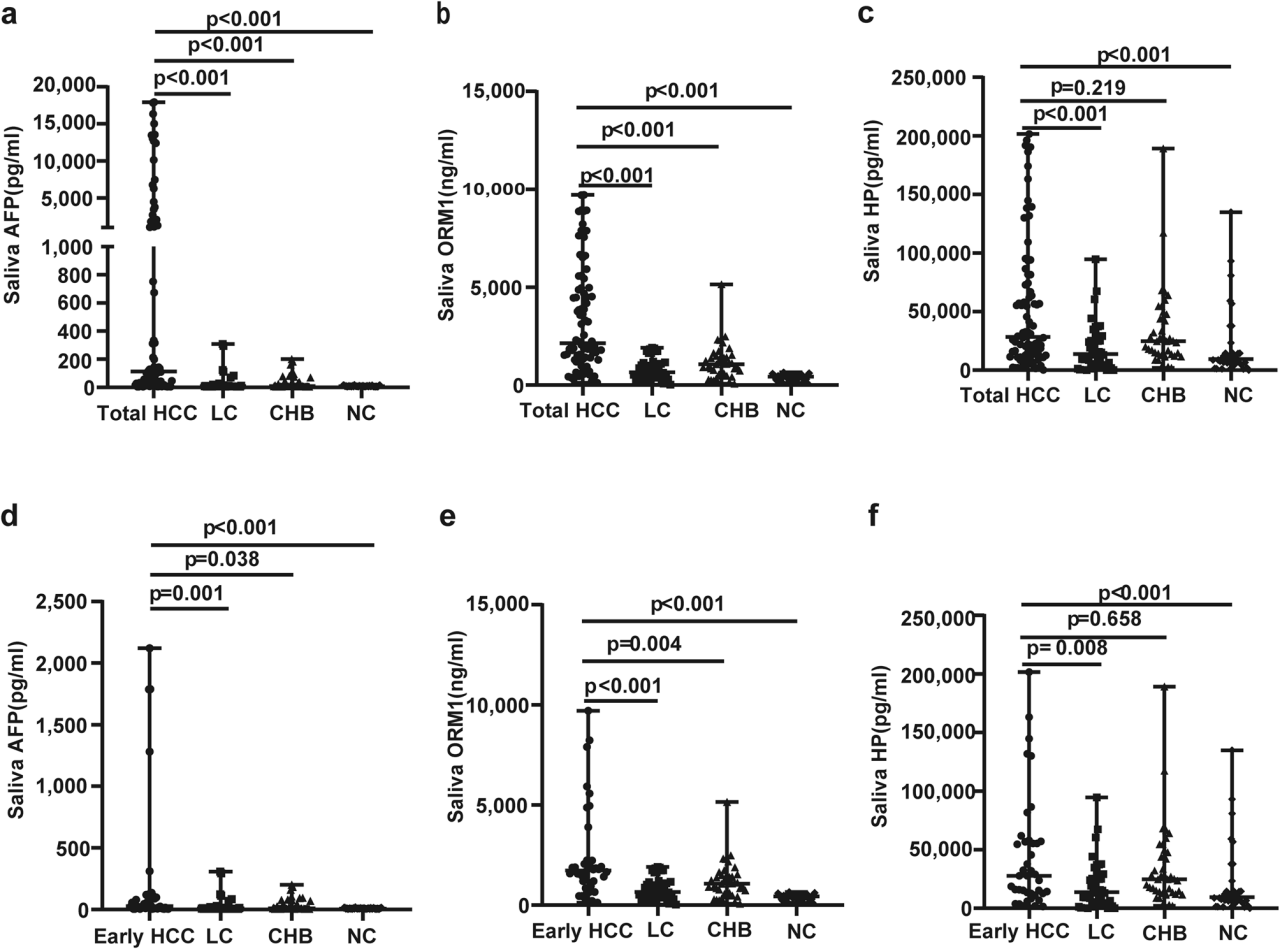
Elisa verification. (**a**) Salivary AFP expression in total HCC, LC, CHB and NC groups. (**b**) Salivary ORM1 expression in total HCC, LC, CHB and NC groups. (**c**) Salivary HP expression in HCC, LC, CHB and NC groups. (**d**) Salivary AFP expression in early HCC, LC, CHB and NC groups. (**e**) Salivary ORM1 expression in early HCC, LC, CHB and NC groups. (**f**) Salivary HP expression in early HCC, LC, CHB and NC groups. *AFP* α-fetoprotein, *HP* Haptoglobin, *ORM1* Orosomucoid1, *HCC* hepatocellular carcinoma, *LC* liver cirrhosis, *CHB* chronic viral hepatitis B, *NC* normal control. Data are shown as median with range.

Quantitative analysis of saliva AFP, ORM1 and HP was performed (Table 4). The level of salivary AFP was significantly higher in the HCC group than in the non-HCC group (*p* < 0.001) (Fig. 3a). Saliva ORM1 levels in the HCC group were significantly higher than in the non-HCC group (*p* < 0.001) (Fig. 3b). Saliva HP expression in the HCC group was significantly higher than in the LC and NC groups (*p* < 0.001), but there was no statistically difference in salivary HP between the HCC and CHB groups (*p* = 0.219) (Fig. 3c). The level of salivary AFP was significantly higher in the early HCC group than in the non-HCC group (LC: *p* = 0.001,CHB:*p* = 0.038,NC: *p* < 0.001) (Fig. 3d).The level of salivary ORM1 was significantly higher in the early HCC group than in the non-HCC group(LC: *p* < 0.001,CHB: *p* = 0.004,NC: *p* < 0.001) (Fig. 3e). The level of salivary HP was higher in the early HCC group than in the non-HCC group, but there was no statistically difference in salivary HP between the HCC and CHB groups (LC: *p* = 0.008, CHB: *p* = 0.658, NC: *p* < 0.001) (Fig. 3f). Other researchers in our group used proteomics to detect elevated expression of AFP and ORM1 in both blood and urine in the HCC group^15,16^. Combined with the above results, we took salivary AFP and ORM1 as target proteins.

**Table 4:** The expressions of Salivary AFP, ORM1 and HP in each group.

	HCC	LC	CHB	NC	Early HCC	Advanced HCC
Salivary AFP (pg/ml)	113(6, 17,922)	9(6, 308)	10(6, 200)	8(6, 16)	22(6,2119)	1081(3,17,922)
Salivary ORM1 (ng/ml)	2143(117, 9716)	657(25, 1912)	1073 (70, 5149)	429 (107, 654)	1723(117,9705)	4029(290,9716)
Salivary HP (pg/ml)	28,390(210,201524)	13,767(156,94718)	24,737(2084,189133)	9452(156,134846)	17,326(1574,201524)	48,522(210,196300)

The quantitative variables of the non-normal distribution were represented by median (range). Abbreviations: *NC* normal control, *CHB* chronic hepatitis B, *LC* liver cirrhosis, *HCC* hepatocellular carcinoma, *AFP* α-fetoprotein, *ORM1* Orosomucoid 1, *HP* Haptoglobin.

### Target proteins and prognosis of HCC

**Figure 4:**
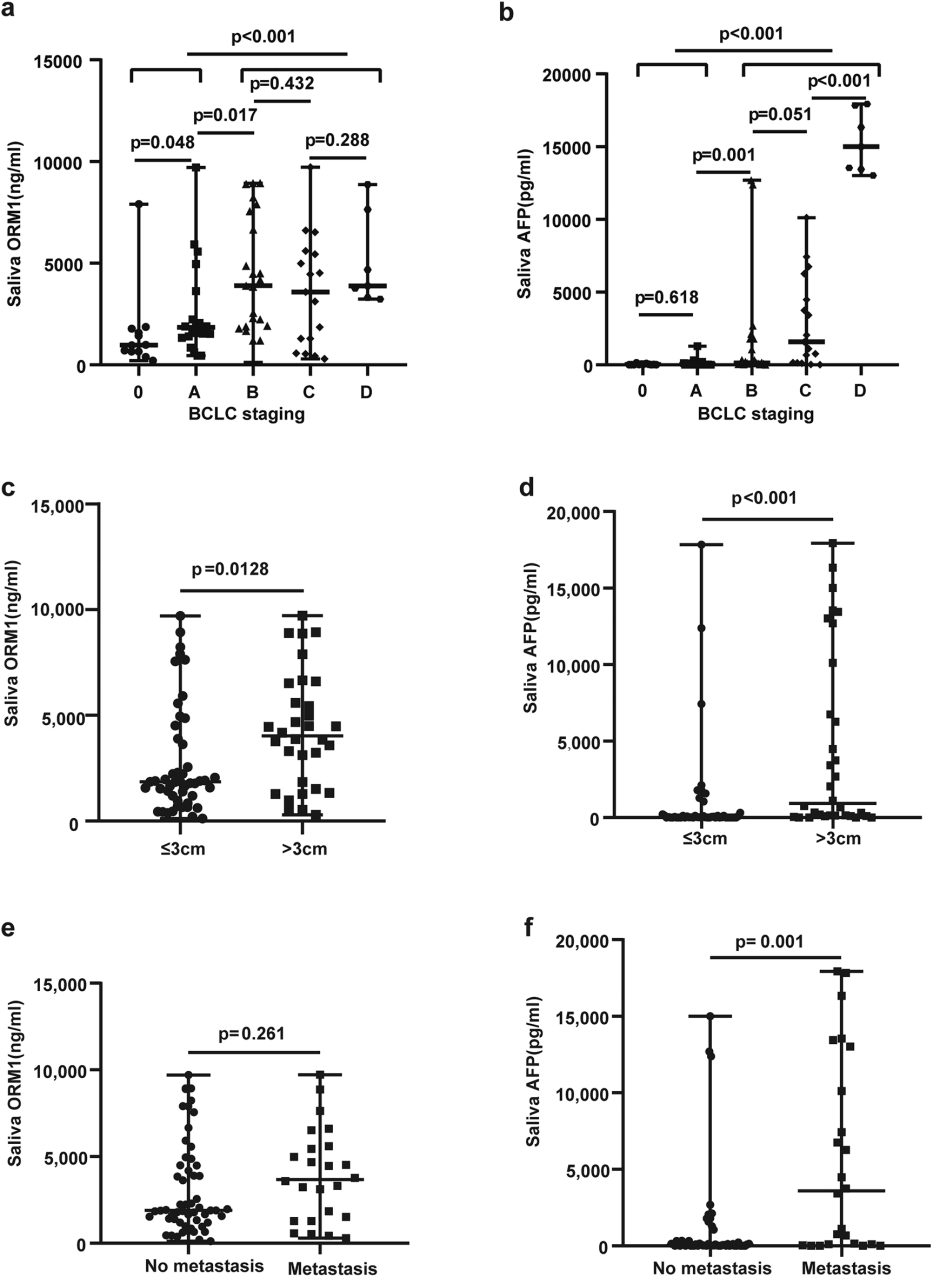
Target proteins and prognosis of HCC. (**a**) Salivary ORM1 expression difference between early and late HCC. (**b**) Salivary AFP expression difference between early and late HCC. (**c**) Salivary ORM1 expression difference between HCC patients with the largest tumor diameter ≤ 3 cm and HCC patients with the largest tumor diameter > 3 cm. (**d**) Salivary AFP expression difference between HCC patients with the largest tumor diameter ≤ 3 cm and HCC patients with the largest tumor diameter > 3 cm. (**e**) Salivary ORM1 expression difference between HCC patients with and without metastasis. (**f**) Salivary AFP expression difference between HCC patients with and without metastasis. *AFP* α-fetoprotein, *HP* Haptoglobin, *ORM1* Orosomucoid1, *HCC* hepatocellular carcinoma. Data are shown as median with range.

Saliva ORM1and saliva AFP were significantly higher in the advanced HCC (BCLC B, C and D) group than in the early HCC (BCLC0 and BCLC A) group (*p* < 0.001) (Fig. 4a and b). There was no statistically significant difference in salivary ORM1 between BCLC 0 and BCLC A, nor between BCLC stage B, C and D. The expression levels of ORM1 and AFP in saliva of HCC patients with the largest tumor diameter > 3 cm were significantly higher than those of HCC patients with the largest tumor diameter ≤ 3 cm (ORM1:*p* = 0.0128,AFP:*p* < 0.001) (Fig. 4c and d). The expression levels of ORM1 in saliva of HCC patients with metastasis were higher than those of HCC patients without metastasis (*p* = 0.261) (Fig. 4e).The expression levels of AFP in saliva of HCC patients with metastasis were significantly higher than those of HCC patients without metastasis (*p* = 0.001) (Fig. 4f).

### Correlation analysis of salivary AFP and serum AFP

**Figure 5:**
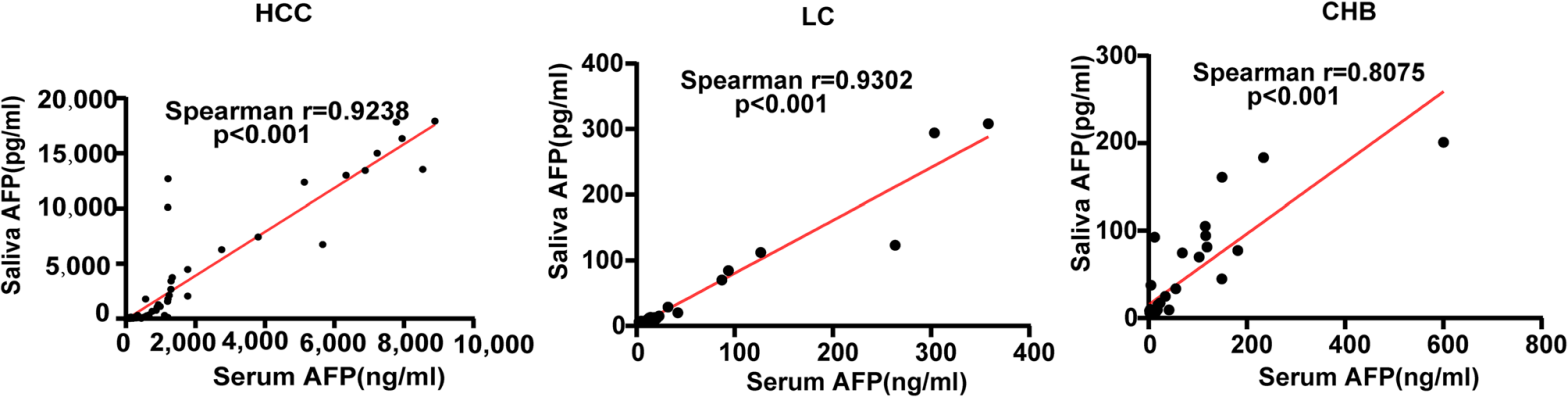
Correlation analysis of salivary AFP and serum AFP. *AFP* α-fetoprotein, *HCC* hepatocellular carcinoma, *LC* liver cirrhosis, *CHB* chronic viral hepatitis B.

We analyzed the correlation between salivary AFP and serum AFP (Fig. 5). The significant correlation was observed in HCC, LC and CHB cases between saliva AFP and serum AFP (*p* < 0.001).

### Diagnostic efficacy of salivary AFP and salivary ORM1

**Figure 6:**
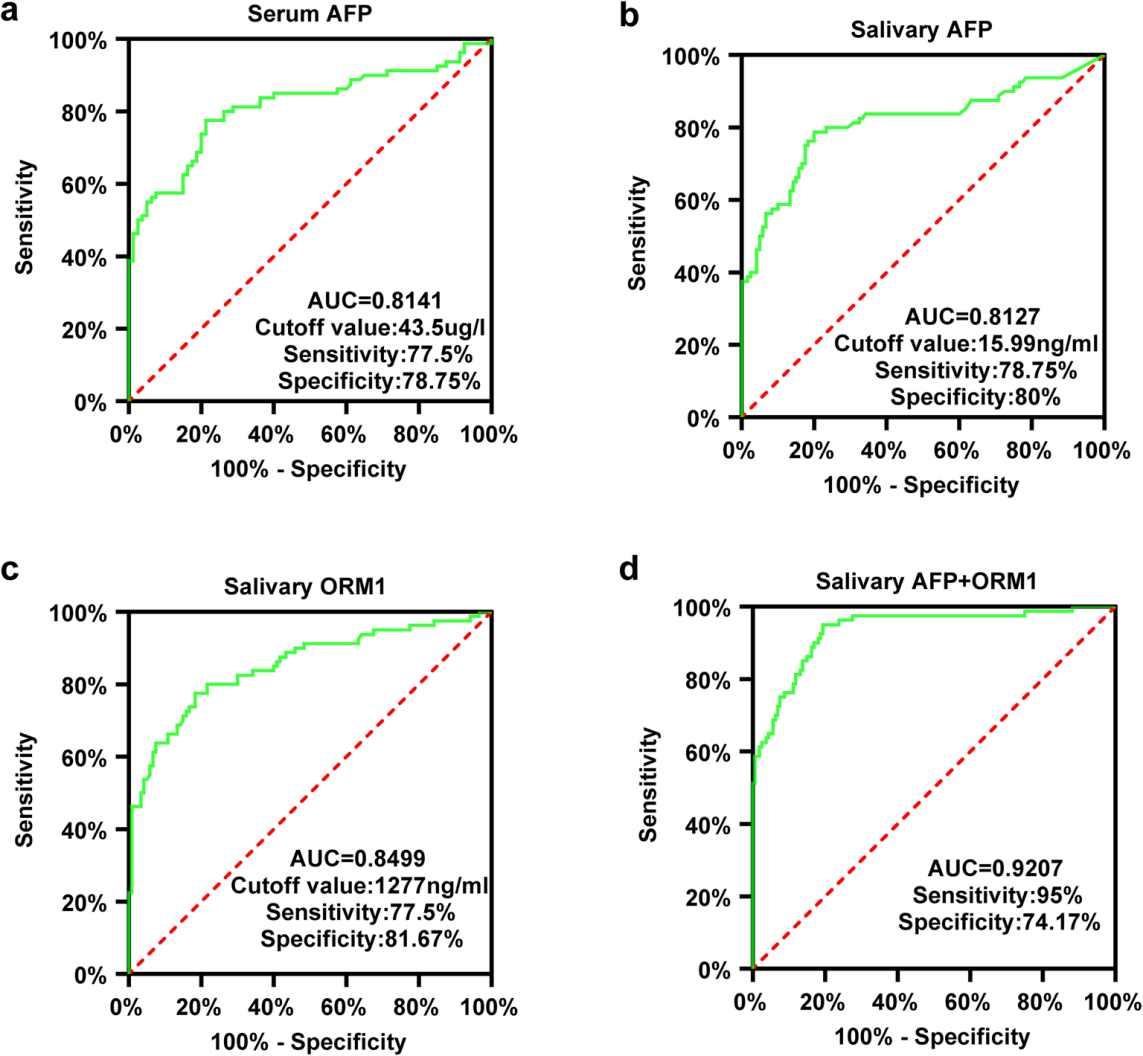
ROC curves. (**a**) The ROC curves of serum AFP in diagnosis HCC. (**b**) The ROC curves of salivary AFP in diagnosis HCC. (**c**) The ROC curves of salivary ORM1 in diagnosis HCC. (**d**) The ROC curves of saliva ORM1 and saliva AFP combination in diagnosis HCC. *AFP* α-fetoprotein, *ORM1* Orosomucoid1, *HCC* hepatocellular carcinoma, *LC* liver cirrhosis, *CHB* chronic viral hepatitis B, *ROC* Receiver operating characteristic.

We plotted ROC curves for serum AFP,salivary AFP, salivary ORM1, and combined salivary AFP + ORM1. The cut-off value was assessed according to the Youden index (sensitivity + specificity − 1). When we took the LC group, CHB group and NC group together as the control group, the AUC of serum AFP was 0.8141 (95% CI: 0.7452–0.8829), with a sensitivity of 75.5% , a specificity of 78.75% , when the cutoff point was set to 43.5 ug/l (Fig. 6a); the AUC of salivary AFP was 0.8127 (95% CI: 0.7455 to 0.8799), with a sensitivity of 78.75% , a specificity of 80% , when the cutoff point was set to 15.99 ng/ml (Fig. 6b); the AUC of salivary ORM1 was 0.8499(95% CI: 0.7724–0.9075), with a sensitivity of 77.5%, a specificity of 81.67%, when the cutoff point was set to 1277 ng/ml (Fig. 6c); the AUC of salivary AFP + ORM1 combination was 0.9207 (95% CI: 0.8825 to 0.9590), with a sensitivity of 95%, a specificity of 74.17%, (Fig. 6d). Salivary ORMI may be a potential biomarker for diagnosis or screening of HCC, and performed much better when combined with salivary AFP.

## Discussion

The current recommendation is that people with liver cancer risk factors be screened for liver cancer every 3–6 months^17^. A high proportion of HCC is detected late in clinical practice due to limitations imposed by surveillance. But liver cancer is a rapidly progressing cancer, with a one-year survival rate of 36 percent after diagnosis^18^.Therefore, it is particularly important to explore an economical and convenient, for both patients and medical staff, method to screen for liver cancer.

In this study, ORM1 was found to be a potential salivary marker for HBV-associated liver cancer. The sensitivity and specificity of salivary ORM1 combined with salivary AFP in the diagnosis of hepatocellular carcinoma reached 95% and 74.17%. Salivary ORM1 was significantly elevated in patients with advanced liver cancer compared to patients with early liver cancer, and in patients with tumors larger than 3 cm in diameter compared to patients with tumors less than 3 cm in diameter.Elevated salivary ORM1 may be a risk factor for poor prognosis in HCC patients.Although there have been studies on salivary proteomics of liver cancer, this study involved studies on salivary markers of liver cancer, liver cirrhosis, hepatitis B and healthy people, and finally found different salivary markers of liver cancer^19^.

AFP is a glycoprotein synthesized in the fetal yolk sac and liver during pregnancy^20^. In HCC patients, AFP can be reactivated to participate in the regulation of liver cancer pathogenesis and the regulation of chemotherapy sensitivity, and is a classic marker of liver cancer^21,22^. AFP remains the most widely used marker in clinical practice, although new and reliable markers have been developed^23^. Serum AFP has a sensitivity of 40–60% and specificity of 80–90% for the diagnosis of hepatocellular carcinoma at a cut-off value of 20 ng/ml^24^. Serum AFP has a sensitivity of 68–87% and specificity of 74–90% for the diagnosis of hepatitis B associated liver cancer^25,26^. Through correlation analysis, we found that there was a significant correlation between saliva AFP and blood AFP, such that saliva AFP expression level could indirectly reflect the blood AFP level, which was consistent with previous research^9,27^. However, AFP may also be elevated in some patients with cirrhosis and chronic viral hepatitis.

Orosomuoid-1(ORM1), also known as α -1-acid glycoprotein 1(AGP1), is a member of the lipid protein family, also known as acute phase proteins, is expressed in inflammatory injury, inflammation, infection and other stress conditions^28,29^. ORM1 has been reported to be associated with a variety of diseases, including: oral diseases , metabolic diseases , autoimmune diseases, cardiovascular and cerebrovascular diseases and other cancers such as lung cancer, bladder cancer, and breast cancer^29^. Ye et al. reported serum ORM1 expression is downregulated in stage I non-small cell lung cancer (NSCLC) patients compared to healthy controls but is upregulated in stage IV patients, ORM1 expression was regulated by TGF-β and mediated by the TGF-β/Smad signalling pathway^30^. Li F et al. reported urinary ORM1 is significantly elevated in urine of bladder cancer patients, compared with controls and benign cases, it may be associated with the inflammatory activation in bladder cancer patients or associated with vascular endothelial cells, especially angiogenically activated blood vessels^31^. Luo Q et al. reported serum ORM1 has been shown to be upregulated in the serum of breast cancer patients, ORM1 expression is associated with proinflammatory factors such as interleukin (IL)-1β, IL-8, and tumor necrosis factor-α ^32,33^. ORM1 is predominantly synthesized in the liver, hepatic diseases may have more influence on its expression, ORM1 can be induced in liver injury and activate the liver cell cycle to achieve liver regeneration^34^. Gu J et al. reported ORM1 can promote tumor growth, ORM1 expression was highly correlated with tumor grade and stage^35^. Current studies on ORM1 expression association with other cancers have focused on blood, urine and cancerous tissue and the level of salivary ORM1 expression in other cancer patients is worthy of further validation. Our current study cannot prove that salivary ORM1 is a specific marker of liver cancer, but our screening and diagnosis of liver cancer mainly focus on patients with chronic liver disease caused by hepatitis B virus infection. Hepatitis B virus infection is one of the major risk factors for liver cancer, it is of great importance to explore a simple method for screening for liver cancer in patients with hepatitis B virus infection. The sensitivity and specificity of serum alpha-1-acid glycoprotein(AGA) in AFP-negative HCC patients were 82% and 90%, and 62% and 82% in HCC patients with elevated AFP^36^. The sensitivity and specificity of serum AFP and AGA in the diagnosis of HCC were 85–89% and 83–90%, respectively^37,38^. We are the first to report that salivary orosomucoid 1 as a biomarker of hepatitis B associated hepatocellular carcinoma.

Saliva is rich in proteins, DNA, RNA and microorganisms, and can be regarded as a biomarker library^39^. Markers of cancer present in the blood can also be found in saliva, and changes in their concentration can be used as biomarkers to detect early cancer or monitor the response to treatment management^40^. Saliva collection is noninvasive, a simple operation, and does not require professional medical personnel. Furthermore, substances in saliva last longer than in blood^41^. Compared to blood and urine, saliva testing can reduce the workload of medical staff, reduce patients' pain, and increase patients' compliance. We found that saliva AFP and saliva ORM1 combination have higher diagnostic efficacy than serum AFP. And we can attempt to develop saliva test paper to screen for HCC high-risk populations and mass screening in areas with high incidences of liver cancer.

However, the number of subjects included in this study is small, so further study is needed to expand the sample size and test our experimental results further. Subsequent studies can also explore ORM1 influence on the prognosis of liver cancer and its mechanism further. The etiology of HCC patients included in this study was hepatitis B virus infection, so HCC caused by other etiologies requires further study. Of course, saliva detection is also affected by oral diseases, and in some cases cannot replace blood tests.

## Conclusion

Salivary ORMI may be a potential biomarker for diagnosis or screening of HCC, and performed much better when combined with salivary AFP. In the future, we can attempt to develop saliva test strips to screen people at high risk for liver cancer.

## Supplementary Information

Supplementary Information.

## Data Availability

The datasets generated and/or analysed during the current study are available from the corresponding author on reasonable request. Proteomics data are available through the Pride Repository (weblink: http://www.ebi.ac.uk/pride/archive/projects/PXD033752, accession number: PXD033752).
