# Supplementary material for: Salivary orosomucoid 1 as a biomarker of hepatitis B associated hepatocellular carcinoma
Source: Sci Rep. 2022 Sep 12;12:15347. doi: 10.1038/s41598-022-18894-2 (PMC9467997; doi:10.1038/s41598-022-18894-2)

Salivary orosomucoid 1 as a biomarker of hepatitis B associated hepatocellular carcinoma

Jiaoxia He[1], Zhongling Zheng[1], Tingting Liu[1], Yupei Ao[1], Yixuan Yang[1], Huaidong Hu[1*]

[1]Department of Infectious Diseases, Key Laboratory of Molecular Biology for Infectious Diseases, Institute for Viral Hepatitis, the Second Affiliated Hospital of Chongqing Medical University, Chongqing 400000, People's Republic of China.

*Corresponding author: Huaidong Hu, E-mail: Huhuaidong@cqmu.edu.cn.

Supplementary Table S1.152 differentially expressed proteins

Number	Protein_ID（uniport）	Gene symbol	Description	Peptides (95%)	HCC : NC	HCC : CHB	HCC：LC	mean ratios	Down or Up regulation
1	Q9Y3C6	PPIL1	Peptidyl-prolyl cis-trans isomerase-like 1	2	2.434933	2.514644	2.281397	2.410325	Up
2	P02771	AFP	Alpha-fetoprotein	11	2.525107	2.466624	2.190237	2.393989	Up
3	O75015	FCGR3B	Low affinity immunoglobulin gamma Fc region receptor III-B	2	1.652728	2.115158	2.089728	1.952538	Up
4	P00738	HP	Haptoglobin	9	1.347725	2.218852	2.192762	1.919779	Up
5	P14780	MMP9	Matrix metalloproteinase-9	25	1.675592	1.62907	1.867495	1.724052	Up
6	Q14019	COTL1	Coactosin-like protein	6	1.866304	1.37119	1.878257	1.70525	Up
7	P05089	ARG1	Arginase-1	10	1.822504	1.393274	1.884372	1.70005	Up
8	O43516	WIPF1	WAS/WASL-interacting protein family member 1	4	1.769751	1.637088	1.648348	1.685063	Up
9	P02763	ORM1	Alpha-1-acid glycoprotein 1	5	1.329538	1.679294	1.857399	1.622077	Up

10	P07737	PFN1	Profilin-1	14	1.756293	1.409054	1.681886	1.615744	Up
11	P00338	LDHA	L-lactate dehydrogenase A chain	16	1.720365	1.41129	1.696693	1.609449	Up
12	P00492	HPRT1	Hypoxanthine-guanine phosphoribosyltransferase	9	1.767694	1.316948	1.684498	1.589713	Up
13	P78417	GSTO1	Glutathione S-transferase omega-1	9	1.692821	1.333049	1.725582	1.583817	Up
14	O94760	DDAH1	N(G)-dimethylarginine dimethylaminohydrolase 1	2	1.969487	1.358008	1.412046	1.579847	Up
15	P22894	MMP8	Neutrophil collagenase	12	1.567222	1.468409	1.691901	1.575844	Up
16	Q2TAA2	IAH1	Isoamyl acetate-hydrolyzing esterase 1 homolog	2	1.621002	1.4287	1.633903	1.561202	Up
17	Q6ZSZ5	ARHGEF18	Rho guanine nucleotide exchange factor 18	2	1.576379	1.651316	1.432885	1.553527	Up
18	P30046	DDT	D-dopachrome decarboxylase	3	1.671673	1.306428	1.665905	1.548002	Up
19	P13796	LCP1	Plastin-2	27	1.619557	1.348721	1.670109	1.546129	Up
20	Q9ULZ3	PYCARD	Apoptosis-associated speck-like protein containing a CARD	7	1.560212	1.347156	1.715193	1.540854	Up
21	P35754	GLRX	Glutaredoxin-1	5	1.709221	1.326525	1.583071	1.539605	Up
22	P52566	ARHGDIB	Rho GDP-dissociation inhibitor 2	11	1.591042	1.356046	1.654111	1.533733	Up
23	O15117	FYB1	FYN-binding protein 1	2	1.538819	1.478342	1.566167	1.527776	Up
24	P50225	SULT1A1	Sulfotransferase 1A1	8	1.391368	1.51965	1.653478	1.521499	Up
25	P25815	S100P	Protein S100-P	3	1.482647	1.360691	1.71291	1.518749	Up

26	Q9H0R4	HDHD2	Haloacid dehalogenase-like hydrolase domain-containing protein 2	4	1.585742	1.341706	1.625159	1.517536	Up
27	P26038	MSN	Moesin	20	1.613644	1.337416	1.601266	1.517442	Up
28	Q14651	PLS1	Plastin-1	2	1.522775	1.43833	1.528969	1.496691	Up
29	P14174	MIF	Macrophage migration inhibitory factor	3	1.779911	1.307765	1.397088	1.494921	Up
30	P30520	ADSS2	Adenylosuccinate synthetase isozyme 2	15	1.551671	1.301466	1.57383	1.475656	Up
31	Q96C19	EFHD2	EF-hand domain-containing protein D2	8	1.541951	1.330635	1.548078	1.473555	Up
32	Q9H8J5	MANSC1	MANSC domain-containing protein 1	2	1.363554	1.419935	1.629395	1.470962	Up
33	Q01518	CAP1	Adenylyl cyclase-associated protein 1	19	1.452661	1.306679	1.594172	1.451171	Up
34	P34059	GALNS	N-acetylgalactosamine-6-sulfatase	3	1.498473	1.33777	1.396389	1.410877	Up
35	Q6P4A8	PLBD1	Phospholipase B-like 1	12	1.380541	1.361218	1.488321	1.410027	Up
36	Q7Z6P3	RAB44	Ras-related protein Rab-44	4	1.313648	1.434466	1.458907	1.40234	Up
37	P01742	IGHV1-69	Immunoglobulin heavy variable 1-69	2	1.493435	1.336299	1.310512	1.380082	Up
38	P09972	ALDOC	Fructose-bisphosphate aldolase C	10	1.476346	1.331318	1.316782	1.374815	Up
39	P49913	CAMP	Cathelicidin antimicrobial peptide	3	1.348133	1.32528	1.396828	1.356747	Up
40	P62993	GRB2	Growth factor receptor-	10	1.380092	1.301272	1.357784	1.346383	Up

			bound protein 2						
41	O94973	AP2A2	AP-2 complex subunit alpha-2	2	1.330447	1.315208	1.311755	1.319137	Up
42	Q9ULC6	PADI1	Protein-arginine deiminase type-1	9	0.731192	0.762952	0.688743	0.727629	Down
43	Q53GQ0	HSD17B12	Very-long-chain 3-oxoacyl-CoA reductase	5	0.714755	0.756893	0.690213	0.72062	Down
44	Q16762	TST	Thiosulfate sulfurtransferase	5	0.755797	0.7535	0.650158	0.719818	Down
45	P63173	RPL38	60S ribosomal protein L38	4	0.737028	0.757955	0.653542	0.716175	Down
46	Q99714	HSD17B10	3-hydroxyacyl-CoA dehydrogenase type-2	7	0.721457	0.766534	0.656343	0.714778	Down
47	Q92747	ARPC1A	Actin-related protein 2/3 complex subunit 1A	5	0.73537	0.752121	0.645588	0.711026	Down
48	P62805	H4C1	Histone H4	8	0.620661	0.759439	0.752681	0.710927	Down
49	P17655	CAPN2	Calpain-2 catalytic subunit	12	0.713291	0.759442	0.651728	0.708154	Down
50	Q08188	TGM3	Protein-glutamine gamma-glutamyltransferase E	32	0.736387	0.764924	0.616479	0.70593	Down
51	P61247	RPS3A	40S ribosomal protein S3a	5	0.723426	0.726509	0.665757	0.705231	Down
52	P55795	HNRNPH2	Heterogeneous nuclear ribonucleoprotein H2	3	0.71183	0.756782	0.645573	0.704728	Down
53	P08708	RPS17	40S ribosomal protein S17	2	0.752453	0.723542	0.637373	0.704456	Down
54	Q9Y2A7	NCKAP1	Nck-associated protein 1	9	0.722899	0.736793	0.647682	0.702458	Down
55	Q969V3	NCLN	Nicalin	2	0.723791	0.758658	0.619971	0.700807	Down
56	O00204	SULT2B1	Sulfotransferase 2B1	5	0.711882	0.713835	0.674889	0.700202	Down
57	P23396	RPS3	40S ribosomal protein S3	11	0.739728	0.761204	0.5994	0.70011	Down

58	P12235	SLC25A4	ADP/ATP translocase 1	2	0.749717	0.74751	0.601431	0.699553	Down
59	P07305	H1-0	Histone H1.0	3	0.666467	0.713715	0.71166	0.697281	Down
60	Q9Y446	PKP3	Plakophilin-3	18	0.758826	0.747164	0.585339	0.69711	Down
61	Q04837	SSBP1	Single-stranded DNA-binding protein, mitochondrial	4	0.685225	0.741346	0.656363	0.694311	Down
62	Q96S59	RANBP9	Ran-binding protein 9	4	0.699658	0.745659	0.629365	0.691561	Down
63	Q8TCJ2	STT3B	Dolichyl-diphosphooligosaccharide--protein glycosyltransferase subunit STT3B	5	0.736266	0.737706	0.600317	0.69143	Down
64	P04843	RPN1	Dolichyl-diphosphooligosaccharide--protein glycosyltransferase subunit 1	12	0.706253	0.734999	0.626998	0.689417	Down
65	P60981	DSTN	Destrin	5	0.671428	0.74914	0.640992	0.687187	Down
66	O60313	OPA1	Dynamin-like 120 kDa protein, mitochondrial	4	0.71902	0.643372	0.692131	0.684841	Down
67	P04792	HSPB1	Heat shock protein beta-1	17	0.753457	0.751953	0.547146	0.684185	Down
68	P15880	RPS2	40S ribosomal protein S2	10	0.716614	0.760084	0.573227	0.683308	Down
69	P62854	RPS26	40S ribosomal protein S26	2	0.746505	0.717128	0.58516	0.682931	Down
70	P36578	RPL4	60S ribosomal protein L4	9	0.73129	0.743487	0.57138	0.682052	Down
71	P21246	PTN	Pleiotrophin	2	0.570246	0.757782	0.71774	0.681923	Down
72	Q02218	OGDH	2-oxoglutarate dehydrogenase, mitochondrial	5	0.660793	0.76047	0.620597	0.68062	Down

73	Q9BZ29	DOCK9	Dedicator of cytokinesis protein 9	3	0.694431	0.738714	0.607259	0.680135	Down
74	P55010	EIF5	Eukaryotic translation initiation factor 5	7	0.679408	0.759821	0.597909	0.679046	Down
75	Q9Y4K1	CRYBG1	Beta/gamma crystallin domain-containing protein 1	19	0.704337	0.733258	0.598273	0.678623	Down
76	Q7Z406	MYH14	Myosin-14	25	0.729962	0.717186	0.584358	0.677169	Down
77	P27482	CALML3	Calmodulin-like protein 3	4	0.69139	0.715922	0.623152	0.676821	Down
78	P43304	GPD2	Glycerol-3-phosphate dehydrogenase, mitochondrial	5	0.684662	0.703656	0.641706	0.676675	Down
79	P46782	RPS5	40S ribosomal protein S5	8	0.705386	0.755718	0.56691	0.676005	Down
80	P45880	VDAC2	Voltage-dependent anion-selective channel protein 2	9	0.687443	0.742797	0.594304	0.674848	Down
81	Q9UNE7	STUB1	E3 ubiquitin-protein ligase CHIP	2	0.717565	0.728561	0.572526	0.672884	Down
82	Q00325	SLC25A3	Phosphate carrier protein, mitochondrial	5	0.670258	0.730253	0.613324	0.671278	Down
83	Q6ZUI0	TPRG1	Tumor protein p63-regulated gene 1 protein	4	0.728568	0.728517	0.553114	0.670066	Down
84	Q9H6S3	EPS8L2	Epidermal growth factor receptor kinase substrate 8-like protein 2	10	0.69579	0.693551	0.620598	0.66998	Down
85	P13073	COX4I1	Cytochrome c oxidase subunit 4 isoform 1, mitochondrial	4	0.666374	0.741759	0.601315	0.669816	Down

86	O15231	ZNF185	Zinc finger protein 185	17	0.721548	0.765655	0.514145	0.667116	Down
87	Q04637	EIF4G1	Eukaryotic translation initiation factor 4 gamma 1	8	0.682963	0.715211	0.60291	0.667028	Down
88	Q9HCY8	S100A14	Protein S100-A14	5	0.685666	0.717787	0.596519	0.666657	Down
89	O75947	ATP5PD	ATP synthase subunit d, mitochondrial	5	0.606111	0.759925	0.62963	0.665222	Down
90	P67936	TPM4	Tropomyosin alpha-4 chain	3	0.712433	0.680492	0.59875	0.663892	Down
91	P27144	AK4	Adenylate kinase 4, mitochondrial	2	0.732505	0.678371	0.579215	0.663364	Down
92	Q08AI8	MAB21L4	Protein mab-21-like 4	11	0.68631	0.715764	0.587952	0.663342	Down
93	Q92817	EVPL	Envoplakin	59	0.746953	0.672041	0.568305	0.662433	Down
94	Q8WWI1	LMO7	LIM domain only protein 7	9	0.726239	0.739841	0.517952	0.661344	Down
95	Q6KB66	KRT80	Keratin, type II cytoskeletal 80	11	0.673057	0.746301	0.561655	0.660338	Down
96	Q5K651	SAMD9	Sterile alpha motif domain-containing protein 9	9	0.701489	0.728864	0.547579	0.659311	Down
97	P14923	JUP	Junction plakoglobin	20	0.703979	0.722191	0.5465	0.657557	Down
98	Q6ZVM7	TOM1L2	TOM1-like protein 2	3	0.684359	0.677855	0.60808	0.656764	Down
99	P46776	RPL27A	60S ribosomal protein L27a	5	0.708037	0.666832	0.591433	0.655434	Down
100	P05387	RPLP2	60S acidic ribosomal protein P2	3	0.686621	0.659341	0.614202	0.653388	Down
101	O95361	TRIM16	Tripartite motif-containing protein 16	10	0.705363	0.633475	0.621145	0.653328	Down
102	P21796	VDAC1	Voltage-dependent anion-selective channel protein 1	10	0.640001	0.707807	0.611307	0.653038	Down
103	P62241	RPS8	40S ribosomal protein S8	5	0.711698	0.690487	0.556759	0.652981	Down

104	Q15126	PMVK	Phosphomevalonate kinase	5	0.713206	0.70038	0.542312	0.651966	Down
105	P08727	KRT19	Keratin, type I cytoskeletal 19	7	0.66699	0.72829	0.560222	0.651834	Down
106	Q96FQ6	S100A16	Protein S100-A16	4	0.728215	0.630336	0.59598	0.651511	Down
107	O95171	SCEL	Sciellin	22	0.713074	0.699871	0.538358	0.650434	Down
108	Q15738	NSDHL	Sterol-4-alpha-carboxylate 3-dehydrogenase, decarboxylating	4	0.703662	0.735439	0.510826	0.649975	Down
109	O60437	PPL	Periplakin	67	0.73177	0.675486	0.537457	0.648238	Down
110	P08729	KRT7	Keratin, type II cytoskeletal 7	4	0.646215	0.72666	0.57171	0.648195	Down
111	O15270	SPTLC2	Serine palmitoyltransferase 2	2	0.756123	0.745274	0.439967	0.647121	Down
112	Q9UGK3	STAP2	Signal-transducing adaptor protein 2	2	0.739955	0.683357	0.517422	0.646911	Down
113	P07355	ANXA2	Annexin A2	18	0.681206	0.717551	0.538662	0.645806	Down
114	Q8WUY1	THEM6	Protein THEM6	2	0.678686	0.676918	0.56505	0.640218	Down
115	P62906	RPL10A	60S ribosomal protein L10a	4	0.650707	0.74637	0.521497	0.639525	Down
116	Q96TA1	NIBAN2	Protein Niban 2	17	0.660301	0.694985	0.558925	0.63807	Down
117	P62851	RPS25	40S ribosomal protein S25	2	0.724269	0.616171	0.570811	0.637084	Down
118	Q13835	PKP1	Plakophilin-1	33	0.712112	0.680687	0.51479	0.635863	Down
119	P15924	DSP	Desmoplakin	117	0.687695	0.705418	0.513318	0.635477	Down
120	P04083	ANXA1	Annexin A1	24	0.700802	0.651518	0.552995	0.635105	Down
121	P32969	RPL9	60S ribosomal protein L9	4	0.692836	0.674135	0.536888	0.63462	Down
122	O95833	CLIC3	Chloride intracellular channel protein 3	6	0.628701	0.730948	0.538862	0.632837	Down
123	P14406	COX7A2	Cytochrome c oxidase	2	0.579102	0.704519	0.608818	0.630813	Down

			subunit 7A2, mitochondrial						
124	P60866	RPS20	40S ribosomal protein S20	2	0.663057	0.704828	0.522899	0.630261	Down
125	Q9UDY2	TJP2	Tight junction protein ZO-2	2	0.672774	0.655775	0.552355	0.626968	Down
126	P02511	CRYAB	Alpha-crystallin B chain	5	0.682504	0.727904	0.470329	0.626913	Down
127	O94832	MYO1D	Unconventional myosin-Id	5	0.612558	0.67336	0.594646	0.626855	Down
128	P13646	KRT13	Keratin, type I cytoskeletal 13	27	0.645715	0.734346	0.490847	0.623636	Down
129	P52943	CRIP2	Cysteine-rich protein 2	3	0.677179	0.661221	0.528797	0.622399	Down
130	P28288	ABCD3	ATP-binding cassette sub-family D member 3	4	0.711397	0.660224	0.491998	0.621207	Down
131	P07476	IVL	Involucrin	8	0.646751	0.690136	0.526231	0.621039	Down
132	P62249	RPS16	40S ribosomal protein S16	7	0.651521	0.731755	0.464378	0.615885	Down
133	P03973	SLPI	Antileukoproteinase	10	0.52309	0.702978	0.618338	0.614802	Down
134	P05388	RPLP0	60S acidic ribosomal protein P0	9	0.62746	0.666866	0.539933	0.61142	Down
135	Q8NEY4	ATP6V1C2	V-type proton ATPase subunit C 2	2	0.634148	0.641672	0.555686	0.610502	Down
136	P51648	ALDH3A2	Aldehyde dehydrogenase family 3 member A2	6	0.561314	0.732763	0.531873	0.60865	Down
137	Q15363	TMED2	Transmembrane emp24 domain-containing protein 2	2	0.632973	0.725187	0.463736	0.607299	Down
138	Q9P0I2	EMC3	ER membrane protein complex subunit 3	2	0.64435	0.65213	0.506571	0.601017	Down
139	Q6ZN66	GBP6	Guanylate-binding protein 6	22	0.696282	0.568716	0.530647	0.598548	Down
140	Q9C002	NMES1	Normal mucosa of esophagus-specific gene 1	2	0.623391	0.626372	0.537204	0.595656	Down

			protein						
141	P19012	KRT15	Keratin, type I cytoskeletal 15	8	0.615327	0.663424	0.494415	0.591055	Down
142	P19013	KRT4	Keratin, type II cytoskeletal 4	40	0.608716	0.67052	0.490925	0.590054	Down
143	Q9BRX8	PRXL2A	Peroxiredoxin-like 2A	5	0.627464	0.633602	0.496416	0.585827	Down
144	P50238	CRIP1	Cysteine-rich protein 1	2	0.575434	0.672195	0.503225	0.583618	Down
145	Q8TD06	AGR3	Anterior gradient protein 3	2	0.519082	0.663697	0.535222	0.572667	Down
146	Q9C075	KRT23	Keratin, type I cytoskeletal 23	3	0.507833	0.686256	0.496605	0.563565	Down
147	Q8N1N4	KRT78	Keratin, type II cytoskeletal 78	24	0.543894	0.630052	0.501987	0.558644	Down
148	O94929	ABLIM3	Actin-binding LIM protein 3	2	0.633982	0.573006	0.447818	0.551602	Down
149	P05787	KRT8	Keratin, type II cytoskeletal 8	4	0.552555	0.614744	0.469718	0.545672	Down
150	P56134	ATP5MF	ATP synthase subunit f, mitochondrial	2	0.505351	0.600319	0.502711	0.536127	Down
151	O95994	AGR2	Anterior gradient protein 2 homolog	2	0.383999	0.537652	0.424869	0.44884	Down
152	Q9UGL9	CRCT1	Cysteine-rich C-terminal protein 1	2	0.421692	0.470476	0.365285	0.419151	Down

Abbreviations: HCC, hepatocellular carcinoma; CHB, chronic hepatitis B; LC, liver cirrhosis; NC, healthy controls. HCC: NC, HCC: CHB, and HCC: LC refer to relative level of protein expression in HCC as compared to NC, CHB, and LC, respectively. 152 proteins in total were identified by at least 2 peptides and a change in abundance beyond the determined cutoff (>1.3 or <0.77) across all the iTRAQ ratios (in duplicates).

supplementary figure

a.AFP for Fig.1a

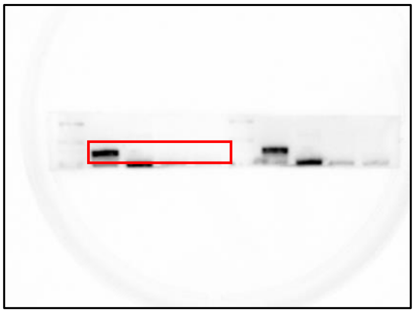

b.ORM1 for Fig.1a

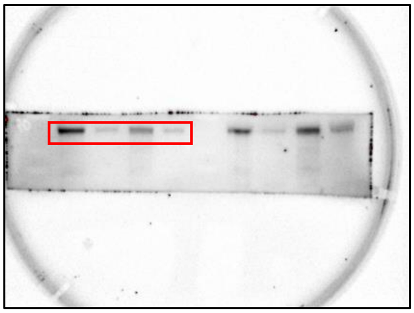

c.HP for Fig.1a

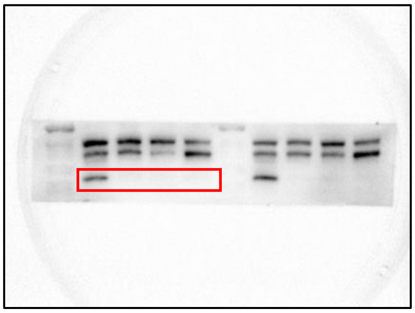

d.MMP9 for Fig.1a

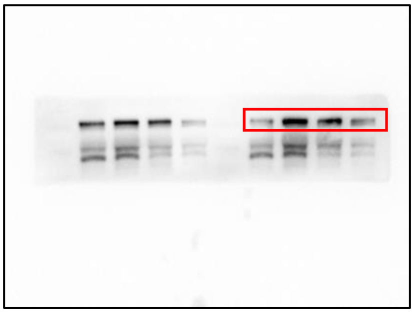

e.ARG1 for Fig.1a

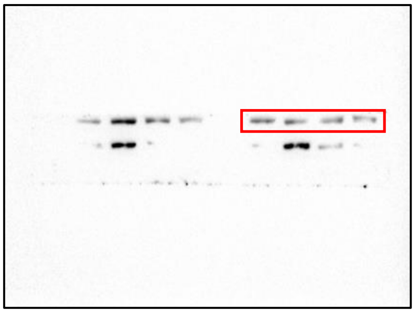

f.FCGR3B for Fig.1a

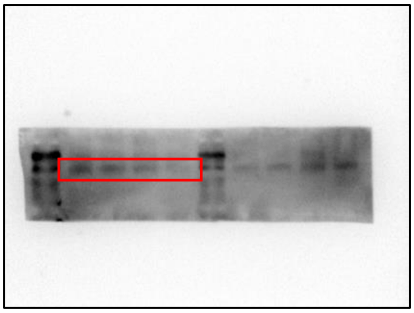

g.COTL1 for Fig.1a

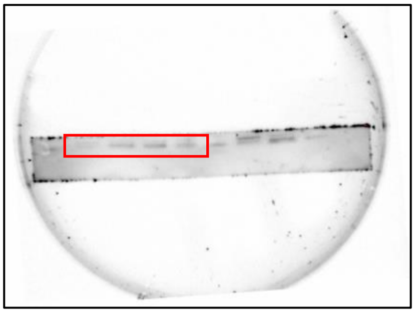

h.GAPDH for Fig.1a

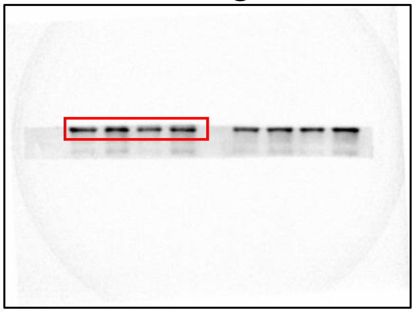

supplementary figure

i.AFP for Fig.1b

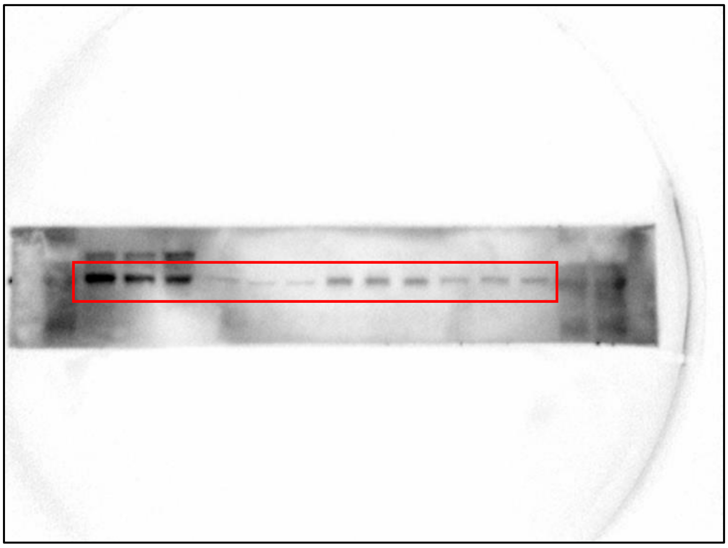

j.ORM1 for Fig.1b

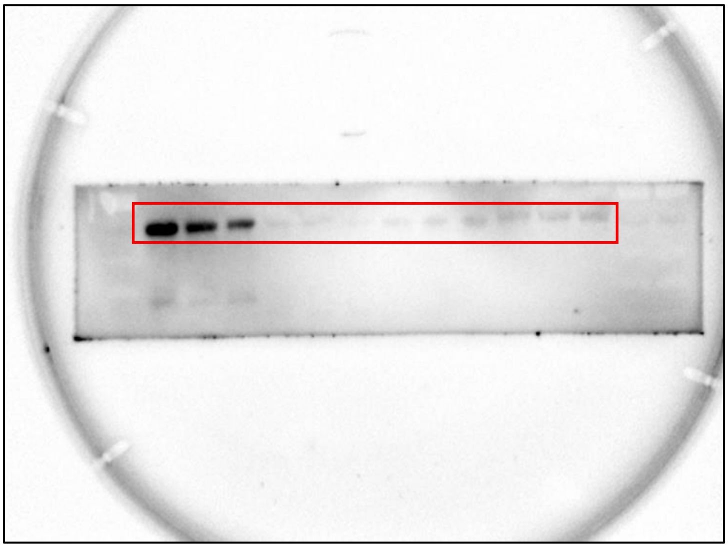

supplementary figure

k.HP for Fig.1b

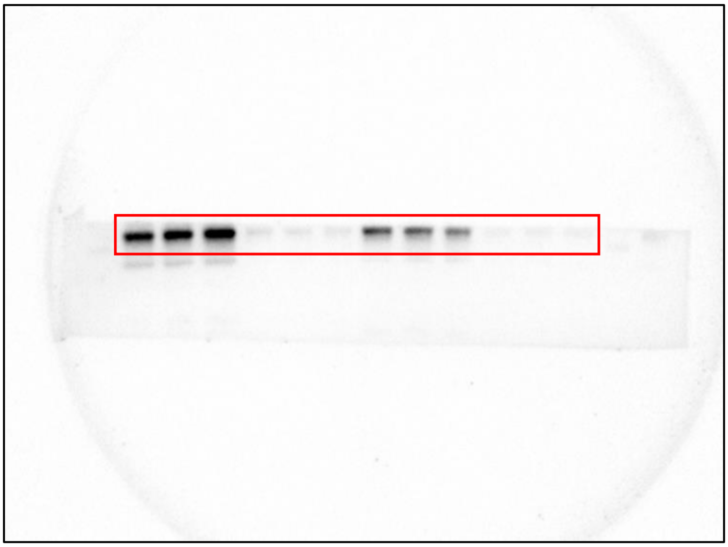

l.GAPDH for Fig.1b

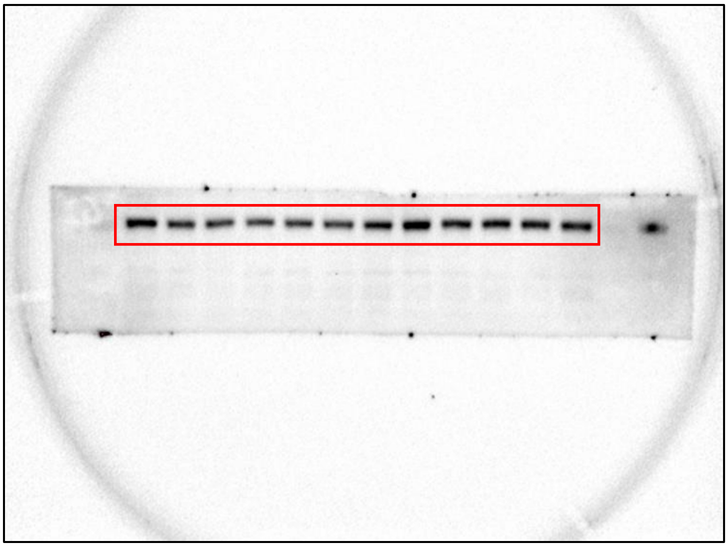

Supplement: Supplementary file 1 — Supplementary Information. [file 41598_2022_18894_MOESM1_ESM.pdf]
